# The Food Niche Overlap and Interspecific Relationship between the Sympatric Tibetan Macaque and Grey Snub-Nosed Monkey

**DOI:** 10.3390/ani13152536

**Published:** 2023-08-06

**Authors:** Li Yue, Cheng Wang, Bingshun Meng, Bo Xie, Heqin Cao, Haijun Su, Mingming Zhang

**Affiliations:** 1 College of Forestry, Research Center for Biodiversity and Nature Conservation, Guizhou University, Guiyang 550025, China; 2 Zhangjiajie Giant Salamander National Nature Reserve Affairs Center, Zhangjiajie 427400, China

**Keywords:** diet composition, grey snub-nosed monkeys, DNA metabarcoding, food niche overlap, Tibetan macaques

## Abstract

**Simple Summary:**

The dietary habits of animals that inhabit the same region can reveal valuable information about their food composition, nutritional strategies, and competition for resources. Analysis of their dietary habits can provide insight into differences in food consumption, thus identifying the potential overlap and competition for resources. In this study, we used DNA metabarcoding to investigate the winter dietary habits of Tibetan macaques and grey snub-nosed monkeys through an analysis of 40 fecal samples. The results showed that Tibetan macaques consumed plants from 117 families and 184 genera, while grey snub-nosed monkeys consumed plants from 109 families and 165 genera. The aim of the research was to assess the winter dietary habits of both monkey species living in the same area, to increase our knowledge of their food preferences and its composition, and to reveal the possible relationship between the overlap of their food niche and interspecific relations, providing useful information for the conservation of the resources in their natural habitat.

**Abstract:**

Assessing the trophic niche and interspecific relationships between related species and determining how the species maintain differences in nutritional niches while coexisting in the same area are important topics in ecological research. Therefore, exploring the mechanism of food resource utilization, competition and coexistence among species distributed in the same region is important. In this study, we used fecal samples and metagenome sequencing technology to study the plant feeding habits and coexistence mechanisms of Tibetan macaques (*Macaca thibetana*) and grey snub-nosed monkeys (*Rhinopithecus brelichi*) within the same area. In the winter of 2020, we collected a total of 40 fecal samples from Tibetan macaques and grey snub-nosed monkeys; of those, 29 samples were considered valid and were analyzed using DNA metabarcoding. The results showed that in winter, Tibetan macaques consumed plants from 117 families and 184 genera, whereas grey snub-nosed monkeys consumed plants from 109 families and 165 genera. Diversity analysis revealed that there was a significant difference in the food composition of Tibetan macaques and grey snub-nosed monkeys. Tibetan macaques had a broader food niche width than grey snub-nosed monkeys at the family and genus levels. In winter, the food niches of Tibetan macaques and grey snub-nosed monkeys almost entirely overlapped (0.99). Our research provides detailed dietary data for Tibetan macaques and grey snub-nosed monkeys and valuable information that can aid in conservation efforts targeting these species.

## 1. Introduction

Competition for resources among sympatric species is a central research topic in community ecology [1]. Since they share habitat resources, sympatric species may only utilize a subset of all resources available in the area [2]. The fundamental concept of resource utilization posits that two species occupying the same n-dimensional niche are likely to experience intense competition [3]. The competitive exclusion principle further indicates that given limited resources, two completely competing species cannot coexist in the long term [4]. Therefore, to minimize overlap with other species, each sympatric species develops a unique niche during the long-term evolutionary process; however, the interplay of ecological requirements and conspecifics leads to complex interspecies relationships, where habitat resource differentiation is a crucial mechanism for the coexistence of sympatric species [3,5,6]. Niche differentiation and interspecific relationships have always intrigued researchers and attracted scholarly attention. To unravel interspecific relationships, some scholars have studied resource allocation related to the ecological niches of fish [7], carnivores [8], ungulates [9], and primates [10,11], among other taxa.

Diet plays a crucial role in shaping the nutritional ecological niche of animals, which devote considerable efforts to foraging for food that provides the energy necessary for their survival [1,12]. However, in winter, food resources become scarce, leading to competitive interactions among coexisting species [13]. Differences in dietary composition among sympatric species arise from the inevitable reduction in competition mechanisms [13]. At the same time, body size also represents a key competitive factor relevant to coexisting species when foraging for food [14]. Furthermore, animal foraging behavior is also influenced by nutrient richness and food palatability.

Traditional diet analysis is commonly conducted using a microscope to inspect the solid remains of food in the feces or stomach contents of animals or through direct observation documenting the food consumed [15]. Microscopic examination heavily relies on the skills of the observer to identify incompletely digested flora and fauna in animal feces or stomach contents. However, it may be challenging to implement direct observation for animals living in densely vegetated areas and those feeding on smaller organisms. The fecal metagenome technique utilized in this study has several advantages over the traditional morphological analysis of fecal contents. First, it is based on the gene level and is therefore unaffected by the external morphology, developmental stage or environmental conditions of the individual [16]. Second, it is efficient and has a high level of accuracy, enabling the classification of mixed samples from various individuals and species simultaneously, thus facilitating the identification of known or new species [17]. Third, the sequencing fragments utilized are usually short and of low quality, making it possible to analyze degraded DNA samples [18]. Last, deep sequencing enhances the detection rate of species with scant representation in mixed or environmental samples [18]. Due to its many advantages, the fecal metagenome has been utilized in dietary studies of various animals, including *Ficedula hypoleuca* [19], European catfish *Silurus glanis* [20], *Chlorocebus pygerythrus* [21], and *Discoglossus scovazzi* [22].

As a vital component of forest ecosystems, primates play a crucial role in maintaining the balance of the ecosystem [23]. To gain a comprehensive understanding of primates, studies are conducted on individual species to determine phylogenetics, migration, feeding patterns, population densities, and less frequently interspecific interactions [23]. However, the most commonly used method by researchers to obtain dietary data for primates is to collect their fecal samples in the wild and then analyze them using DNA metabarcoding. Previous studies have employed DNA metabarcoding technology to obtain comprehensive dietary data for species such as *Macaca fascicularis* [24], *Presbytis femoralis* [25], and lemur species [26]. By analyzing the diet data, we are able to enhance our understanding of the dietary diversity and variations among primate species and investigate their adaptation to environmental changes and threats in greater depth [27].

Fanjing Mountain is a critical area for the conservation of biodiversity and a crucial habitat for protected subtropical forest species in the upper reaches of the Yangtze River [28,29]. The area is inhabited by five species of nonhuman primates, including Tibetan macaques (*Macaca thibetana*) and grey snub-nosed monkeys (*Rhinopithecus brelichi*) ([30], pp. 444–445). Tibetan macaques, also known as red-faced monkeys, are classified as second-class protected animals in China and are widely distributed in central and southeastern China, including 49 counties and cities in Guizhou Province, China, such as Songtao, Yinjiang, Jiangkou, and Chishui [31]. Grey snub-nosed monkeys, also known as white shouldered snub-nosed monkeys or grey golden monkeys, are endemic to Guizhou, China [32], are critically endangered according to The International Union for Conservation of Nature Red List (IUCN Red List), and are listed as a first-class protected animal in China [33]. Fanjing Mountain is the sole global habitat of grey snub-nosed monkeys [34]. Current population data indicate that there are only approximately 700 grey snub-nosed monkeys remaining in the wild [35]. Scholars studying the population size, ecology, behavior, distribution, and diet of grey snub-nosed monkeys have provided qualitative information that can support the more efficient protection and management of this critically endangered species [36,37,38]. In the Fanjing Mountain Nature Reserve, Tibetan macaques primarily reside in evergreen broad-leaved forests and in evergreen and deciduous broad-leaved mixed forests at elevations between 400 and 1900 m. On the other hand, grey snub-nosed monkeys inhabit the evergreen broad-leaved mixed forests and the deciduous broad-leaved forests at elevations between 1500 and 2200 m, and their home range overlaps with that of Tibetan macaques [31]. Grey snub-nosed monkeys have a narrow distribution and a small population, whereas Tibetan macaques exhibit a more consistent population over time and space [39]. The home range of grey snub-nosed monkeys, which have a small population, is confined; however, Tibetan macaques have a wider home range than their grey snub-nosed counterparts, which allows them to acquire more food resources. The highly complex topography of Fanjingshan Mountain gives rise to a variable regional microclimate that results in a diverse ecological environment, with distinct forest community types present even in small, localized areas ([30], p. 3). Based on these facts, our research aims to address two main questions: (1) Are there any variations in the dietary composition between Tibetan macaques and grey snub-nosed monkeys? (2) Is there a distinct separation of the nutritional niche between Tibetan macaques and grey snub-nosed monkeys? Ultimately, we hypothesized the following: (1) there are differences in the food composition of Tibetan macaques and grey snub-nosed monkeys, and (2) there is a divergence in the food niches of Tibetan macaques and grey snub-nosed monkeys.

Using a DNA metagenomic approach, we studied the resource utilization and diets of Tibetan macaques and grey snub-nosed monkeys in the ecologically unique region of the Fanjing Mountains. Our aims, through the analysis of fecal samples of sympatric Tibetan macaques and grey snub-nosed monkeys, were (i) to understand their feeding preferences, (ii) to reveal feeding differences between them, and (iii) to identify food niche overlap and interspecific relationships.

## 2. Materials and Methods

### 2.1. Study Area and Fecal Collection

The Fanjing Mountain National Nature Reserve (FJMR) is located at 27°49′50″ to 28°1′130″ N, 108°45′55″ to 108°48′30″ E, covering an area of 419 km^2^. Fanjing Mountain is the central peak of the Wuling Mountain range in the transition zone from the Yunnan Guizhou Plateau to the hills of western Hunan. The FJMR is one of the areas of highest protection priority in the forest biodiversity protection priority area in the upper reaches of the Yangtze River [29]. The complex topography and landform cause a changeable regional microclimate, leading to the emergence of different types of forest communities in some small areas [40]. The reserve is rich in animal and plant resources, with more than 210 species of rare and globally threatened wild plants, such as *Davidia involucrata* and *Abies fanjingshanensis*. There are more than 110 species of rare and globally threatened wild animals, such as *Viverricula indica*, *Syrmaticus ellioti* and *Capricornis milneedwardsii*.

**Figure 1:**
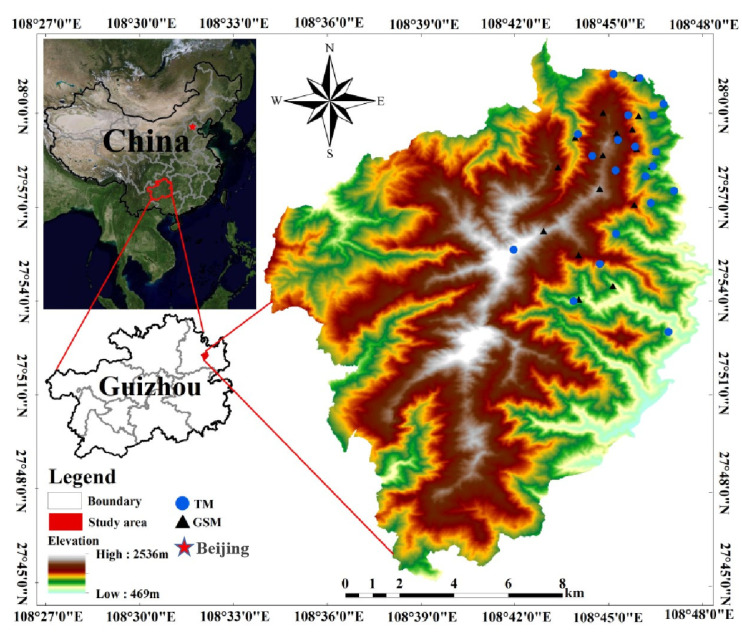
Feces sampling points for the study of grey snub-nosed monkeys and Tibetan macaques in Fanjing Mountain National Nature Reserve (GSM is an abbreviation for grey snub-nosed monkeys, TM is an abbreviation for Tibetan macaques).

We conducted a field study in January 2020, collecting fresh fecal samples from populations of Tibetan macaques and grey snub-nosed monkeys located in the northern FJMR (Figure 1). During sampling, disposable medical gloves were worn to place the samples into 100 mL sterile centrifuge tubes, which were then labeled and placed inside a self-sealing bag, and the GPS location, habitat type and other information were recorded. To avoid the cross-contamination of samples, the gloves were changed every time a sample was taken. The fecal samples were soaked in 95% ethanol on the day of sampling, the ethanol was then poured out after soaking for 24 h, and the samples were transferred to silica gel for drying and preservation. The samples were returned to the laboratory and stored in a special low-temperature freezer at −80 °C.

### 2.2. DNA Extraction

The total DNA of the fecal samples was extracted using the 2CTAB/PCI method [41]. Each DNA extraction included approximately 100 mg of the outer surface for the molecular identification of the species. The confirmed fecal samples from Tibetan macaques and grey snub-nosed monkeys were homogenized, and 100 mg of homogenous feces was used for DNA extraction and molecular dietary analysis [42].

### 2.3. PCR Amplification for Fecal Species Identification

Fecal mitochondrial DNA 16S rRNA fragments were amplified using the following primers Z1aF: 5′-ATGTCACCACCAACAGAGACTAAAGC-3′; hp2R:5′-CGTCCTTTGTAACGATCAAG-3′ [43]; COIintF: 5′-GGWACWGGWTGAACWGTWTAYCCYCC-3′ [44]; COIjgHCO2198: 5′-TANACYTCNGGRTGNCCRAARAAYCA-3′ [45]. The PCR amplification procedure was as follows: predenaturation at 95 °C for 5 min, denaturation at 95 °C for 30 s, annealing at 60 °C for 30 s, 30 cycles of extension at 72 °C for 40 s, and final extension at 72 °C for 7 min. After the reaction, 3 µL PCR products were used for 1% agarose gel electrophoresis to confirm the PCR-amplified fragments. The spliced sequence file was compared with the NCBI nucleic acid database data using the NCBI Blast program (https://blast.ncbi.nlm.nih.gov/Blast.cgi, accessed on 1 July 2023), and the species producing the fecal sample was identified when the similarity of the sequences was over 98%.

During the field survey at the FJMR, a total of 40 fecal samples were collected from Tibetan macaques and grey snub-nosed monkeys. However, after DNA extraction and PCR amplification, it was found that 11 samples did not yield any food data. Therefore, we considered these 11 samples to be invalid. We obtained 29 samples that provided food data, including 18 from Tibetan macaques and 11 from grey snub-nosed monkeys (Table 1).

### 2.4. Amplicon Sequencing

Double-ended sequencing was performed using the Illumina HiSeq X Ten system with a read length of 150 bp at each end. The fecal DNA was purified using the AxyPrep DNA Gel Recovery Kit and Shanghai Personal Biotechnology Co. Ltd. sequenced the purified PCR products. After the splicing, quality control, deduplication, filtering, and chimera removal steps, the reads obtained via sequencing were divided into operational taxonomic units (OTUs) according to a classification level of 98% similarity, and OTU cluster analysis and species taxonomic analysis were performed according to the sample information [46]. Sequence species identification principles included the following: (1) when the sequence comparison results were consistently ≥ 98% and corresponded to only a single species, the sequence was considered to come from that species; (2) when the sequence comparison results were consistently ≥ 98% and corresponded to multiple species, the local undivided species were excluded, and if there were still multiple species, the identification results were recorded as the lowest taxonomic unit covering these species; (3) when the highest recorded consistency of the sequence comparison results was ≥95%, the identification results were recorded as the lowest taxonomic unit covering these species; (4) when the consistency of the sequence comparison result was <95%, the sequence could not be determined and was recorded as “unknown”; and (5) sequences with differences ≤2 were merged [42,46,47].

### 2.5. Food Niche Overlap Analysis

Relative read abundance (*RRA*) is the abundance of sequences in a food group as a percentage of the total food sequence in a valid sample and is used to reflect the relative biomass [48]. Therefore, we used *RRA* to measure the amount of food eaten by Tibetan macaques and grey snub-nosed monkeys [49]. The calculation formula is as follows:

$${RRA}_{i}=\frac{1}{S}\sum_{j=1}^{S}\frac{N_{ij}}{\sum_{i=1}^{T}N_{ij}}\times 100\%$$

where *S* represents the total number of valid samples, *T* represents the number of food groups eaten, and *N_i,j_* represents the number of sequences of food group *i* in sample *j*.

The food overlap between Tibetan macaques and grey snub-nosed monkeys was calculated using the food niche width index and the Schoener food overlap index [47,50]; the calculation formulas are as follows:

Food niche width index:

$$B=1/\sum P_{i}{{}^{2}}_{}$$

where *P_i_* represents the proportion of plants in a certain family/genus to the total number of all plant families/genera;

Schoener food overlap index:

$$D_{ij}=1-0.5\left(\sum \left|\left.P_{ik}-P_{jk}\right|\right.\right)$$

where *i* and *j* denote endemic plants eaten by Tibetan macaques and grey snub-nosed monkeys, respectively, and *k* denotes plants eaten by Tibetan macaques and grey snub-nosed monkeys. *P_ik_* and *P_jk_* represent the proportion of endemic plants eaten by Tibetan macaques and grey snub-nosed monkeys, respectively. *D_ij_* values are between 0 (no overlap) and 1 (complete overlap).

### 2.6. Statistical Analysis

Data were analyzed using IBM SPSS Software v.23. Alpha diversity (Chao1 index, Shannon index, Simpson index and richness index) was calculated using R (4.2.1) software to measure the species abundance and diversity of samples. The nonparametric Kruskal-Wallis rank-sum test for independent samples was used to analyze significant differences in alpha diversity between Tibetan macaques and grey snub-nosed monkeys. Based on the unweighted UniFrac distance, a similarity analysis was performed to test the significance of differences between groups, and a master coordinate analysis plot was generated.

## 3. Results

**Figure 2:**
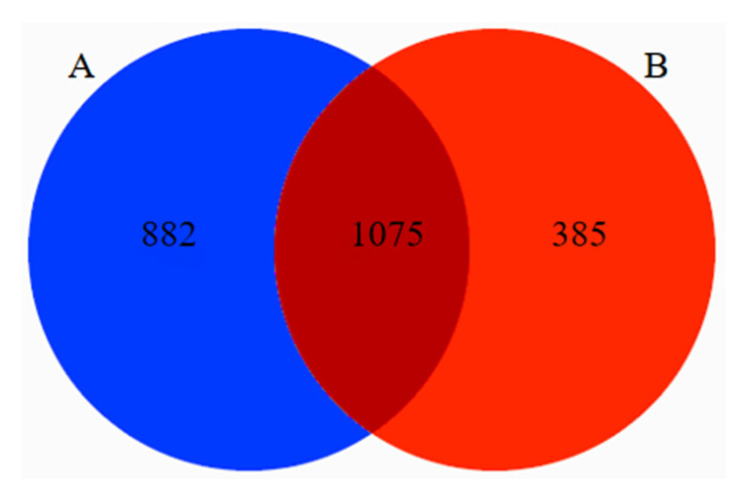
Venn diagram of OTUs between Tibetan macaques (A) and grey snub-nosed monkeys (B) for composition of food contained in the feces sample; the intersection between (A) and (B) represents the number of OTUs shared by Tibetan macaques and grey snub-nosed monkeys. (OTU, which stands for Operational Taxonomic Unit, is a method used in microbial ecology and biodiversity research. It involves grouping similar genetic data sequences together to represent a taxonomic unit. This method is commonly employed when the exact species or taxonomic identity is unknown or difficult to determine).

A total of 3,757,544 original sequences of the target fragment, with a band size of 420 bp, were obtained in 29 fecal samples from Tibetan macaques and grey snub-nosed monkeys. A total of 2,743,662 valid sequences were obtained through primer removal, splicing, mass filtration, deduplication, chimera removal, and clustering of the reads, of which 1,769,343 were from Tibetan macaques and 974,279 were from grey snub-nosed monkeys. The number of sequences obtained in the Tibetan macaques and grey snub-nosed monkeys samples ranged from 63,680 to 119,768, and from 74,853 to 108,268, respectively. The average number of effective sequences obtained from the Tibetan macaques and grey snub-nosed monkeys samples was 98,297 and 88,571, respectively, with an average length of 202 bp. There were 1067 and 1060 OTUs identified in the Tibetan macaques and grey snub-nosed monkeys samples, respectively, clustered with a similarity of 98%. Sorted from 29 samples, a total of 1075 OTUs accounted for 45.9% of the total, 882 OTUs from Tibetan macaques accounted for 37.7% of the total, and 385 OTUs from grey snub-nosed monkeys accounted for 16.4% of the total (Figure 2).

### 3.1. Food Composition of Tibetan Macaques and Grey Snub-Nosed Monkeys

**Figure 3:**
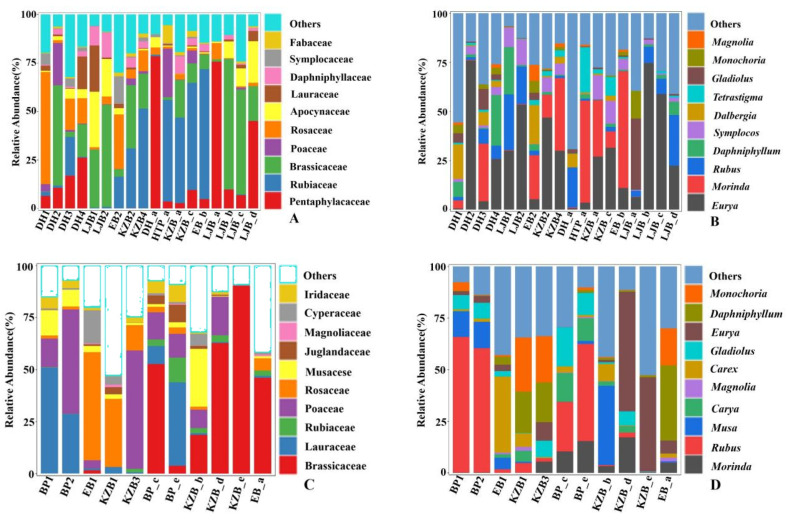
The plant food sources of Tibetan macaques and grey snub-nosed monkeys are among the top 10 in terms of abundance within their respective plant families and genera. (**A**,**B**) are at the family and genus levels of Tibetan macaques, and (**C**,**D**) are the families and genera foraged by grey snub-nosed monkeys, respectively.

A total of 63 orders were identified in 29 fecal samples. A total of 117 families and 184 genera were identified in the fecal samples of Tibetan macaques. At the family level, the preponderant food composition, accounting for more than 1% of the total, was Pentaphylacaceae (3.26%), Rubiaceae (2.99%), Brassicaceae (2.64%) and Poaceae (1.28%) (Figure 3A), and at the genera level, it was *Eurya* (3.25%) and *Morinda* (1.27%) (Figure 3B); 165 genera of 109 families were identified in samples from grey snub-nosed monkeys (Attached Table 1), of which the families that were fed on and accounted for more than 1% of the total were Brassicaceae (2.60%), Lauraceae (1.59%) and Rubiaceae (1.25%) (Figure 3C), and the genera was *Morinda* (1.19%) (Figure 3D).

### 3.2. Analysis of Food Diversity in Tibetan Macaques and Grey Snub-Nosed Monkeys

**Figure 4:**
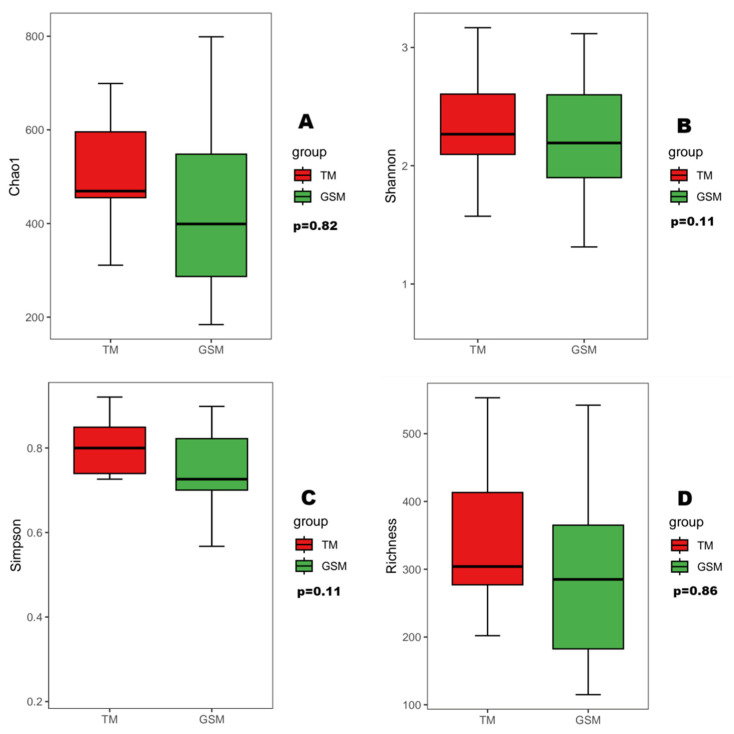
Alpha diversity, including Chao1 (**A**), Shannon (**B**), Simpson (**C**) and Richness (**D**) indices, between Tibetan macaques and grey snub-nosed monkeys. (GSM is an abbreviation for grey snub-nosed monkey, TM is an abbreviation for Tibetan macaque).

**Figure 5:**
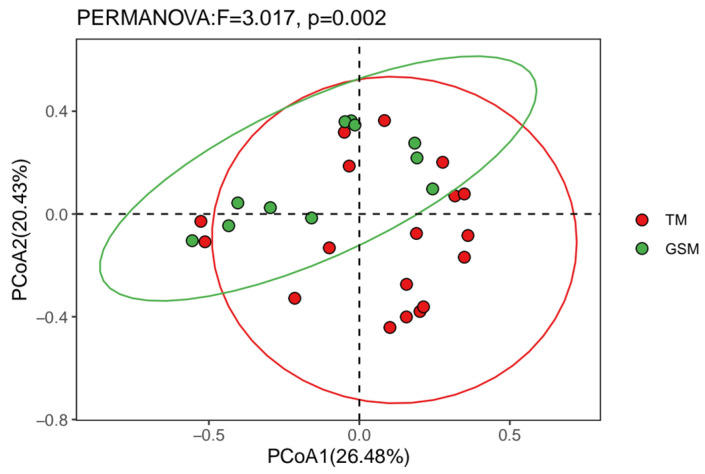
Beta diversity of Tibetan macaques and grey snub-nosed monkeys. (GSM is an abbreviation for grey snub-nosed monkey, TM is an abbreviation for Tibetan macaque).

The α diversity index showed that there was no significant difference in the Chao1, Shannon, Simpson and richness indices between Tibetan macaques and grey snub-nosed monkeys (*p* > 0.05) (Figure 4). PERMANOVA-based β diversity analysis showed that there were significant differences in the diets of Tibetan macaques and grey snub-nosed monkeys (*p* < 0.05) (Figure 5).

### 3.3. Difference Analysis of Food in Tibetan Macaques and Grey Snub-Nosed Monkeys

**Figure 6:**
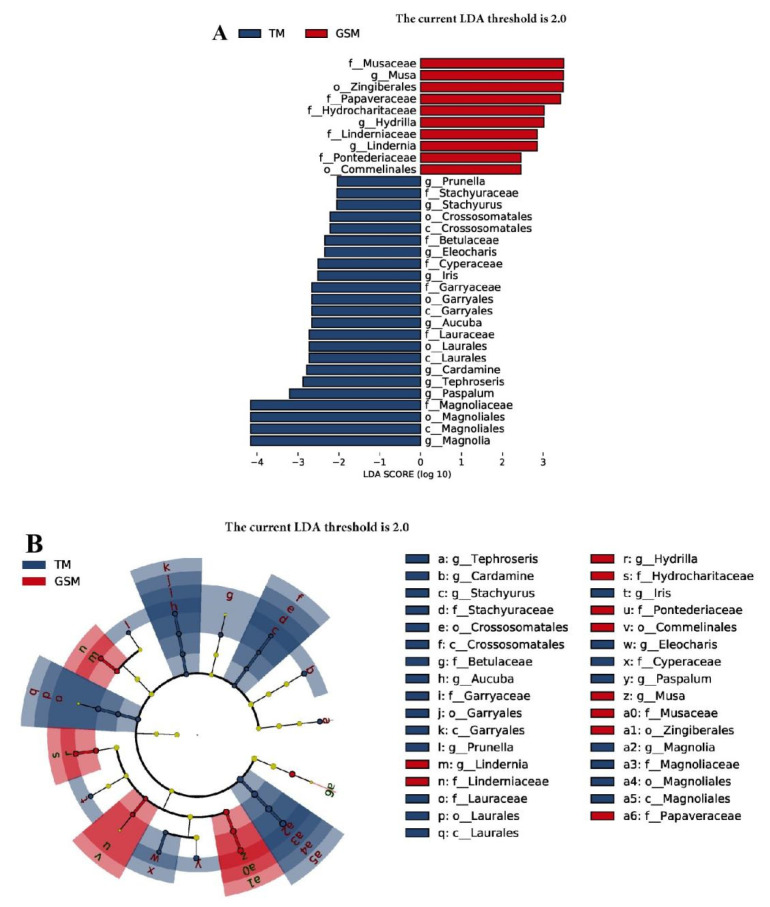
Linear discriminant analysis (LDA) integrated with effect size (LEfSe). (**A**) The differences in abundance between Tibetan macaque and the grey snub-nosed monkey groups. (**B**) Cladogram indicating the phylogenetic distribution of plants correlated with the Tibetan macaque and grey snub-nosed monkey groups (GSM is an abbreviation for grey snub-nosed monkey, TM is an abbreviation for Tibetan macaque).

The linear discriminant analysis effect size (LEfSe) showed that 33 plant compositions in the diets of Tibetan macaques and grey snub-nosed monkeys had discriminant characteristics. In the food composition of grey snub-nosed monkeys, there were five families and three genera of plants that differed from Tibetan macaques, with the family levels Musaceae, Papaveraceae, Hydrocharitaceaey, Linderniaceae and Pontederiaceae, and the genera levels *Musa*, *Hydrilla* and *Lindernia*. Among the dietary compositions of Tibetan macaques, there were 6 families and 8 genera that differed from grey snub-nosed monkeys, including the families Caryophyllaceae, Betulaceae, Platypodaceae, Lauraceae and Magnoliaceae, and the genera *Prunus*, *Spinosaurus*, *Ficus*, *Prunus*, *Astragalus*, *Cardamom*, *Viburnum* and *Pods* (Figure 6A); there were significant differences in nine genera, including *Tephroseris*, *Cardamine* and *Stachyurus*, while the grey snub-nosed monkeys samples had significant differences in three genera, namely *Lindernia*, *Hydrilla* and *Musa* (Figure 6B).

### 3.4. Food Niche Overlap of Tibetan Macaques and Grey Snub-Nosed Monkeys

**Figure 7:**
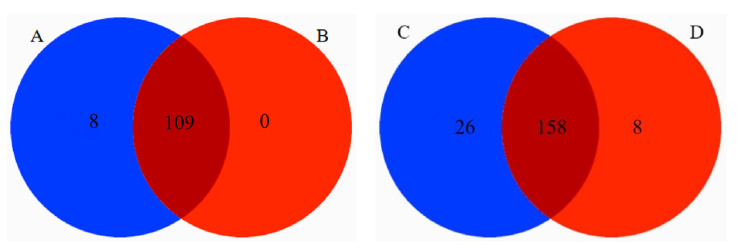
Tibetan macaque and grey snub-nosed monkey unique food with shared food Venn diagram. (A) is a family specific to Tibetan macaques, (B) is a family specific to grey snub-nosed monkeys, (C) is a genus specific to Tibetan macaques, (D) is a genus specific to grey snub-nosed monkeys.

There were 8 families and 26 genera of endemic plants in the diet of Tibetan macaques, while there were 8 genera of endemic plants in the diet of grey snub-nosed monkeys. There were jointly 158 genera and 109 families in the diets of Tibetan macaques and grey snub-nosed monkeys (Figure 7).

At the family and genus levels, the food niche width of Tibetan macaques was wider than that of grey snub-nosed monkeys. At the family and genus levels, the food overlap of Tibetan macaques and grey snub-nosed monkeys reached almost 1 (Table 2).

## 4. Discussion

### 4.1. Diet of the Two Primates

In this study, the winter diets of Tibetan macaques and grey snub-nosed monkeys, two primates with overlapping distributions at Fanjingshan, were investigated. The dietary preferences of the two primates can be inferred from their respective diets. We found that Tibetan macaques at Fanjingshan had a more varied diet than those found in the Anhui province of China [12]. Our study showed that the dietary composition of grey snub-nosed monkeys differed from the results of previous research on this topic [35,38,51]. Nonetheless, given the disparity in research methods, it is not possible to make a direct comparison of the plant species consumed in each primate study. The differences between these results and those of our study are likely related to differences in research methods. Previous research has shown that fecal samples can be effectively employed for metagenomic sequencing, resulting in the rapid acquisition of dietary data in animals with complex diets [25]. Consequently, the metagenomic analysis of fecal samples has emerged as a popular method in animal dietary research, particularly for species with complex dietary patterns such as fish [7], primates [52,53], carnivores [8], and ungulates [9].

Liu et al. [12] used quick scanning sampling to study Tibetan macaques in Huangshan city, Anhui Province, and found that Tibetan macaques fed on the plants of 23 families and 31 genera in winter. Nie et al. [51] reported that grey snub-nosed monkeys feed on 49 genera of plants in 25 families; Guo et al. [54] reported that grey snub-nosed monkeys feed on 31 families and 51 genera; Xiang et al. [50] reported that the diet of grey snub-nosed monkeys contains 107 plants from 28 families and 58 genera, and includes mainly mature leaves and buds in winter. However, through the use of the DNA metabolism barcoding analysis method, we found that the diet of Tibetan macaques consists of food items from 117 families and 184 genera, while the diet of grey snub-nosed monkeys includes food items from 109 families and 165 genera. This is slightly different from the results of this paper. The feeding behaviors of Tibetan macaques and grey snub-nosed monkeys have been examined by researchers using the instantaneous scanning sampling method. Nonetheless, this method is impacted by both internal and external factors at the study site, such as the geographical location of observation points and vegetation distribution, leading researchers to record solely the tall trees and shrubs the Tibetan macaques and grey snub-nosed monkeys consume, thus limiting observations in areas with dense vegetation and low herb coverage. DNA metabarcoding analysis provides a more comprehensive approach than direct observation in food studies [55].

By employing DNA metagenomic sequencing, this study benefited from efficient and accurate deep sequencing, resulting in the acquisition of more comprehensive dietary data than in previous studies. However, identifying specific edible plant materials consumed by Tibetan macaques and grey snub-nosed monkeys remains challenging, despite the effectiveness of DNA metagenomic sequencing for accurately deep-sequencing fecal contents. To comprehensively study animal diets, the optimal approach in future research is to combine direct observation with DNA microbiome sequencing. This will enable the comprehensive acquisition of diet data, which is essential to improving wildlife protection.

### 4.2. Food Diversity in the Two Primates

The alpha diversity analysis of the diets of Tibetan macaques and grey snub-nosed monkeys indicated no significant difference between their diets. However, beta diversity analysis and linear discriminant analysis effect size (LEfSe) suggested that Tibetan macaques consume a wider variety of food than grey snub-nosed monkeys.This is consistent with our hypothesis (1). The availability of plant species that animals consume is primarily limited by the distribution of vegetation in their habitat [56]. This view is supported by studies on *Indri indri* [57], Macaca [58] and *Cercopithecus mitis kandti* [59], which also adapt their diets to available food resources in their environment. Tibetan macaques are distributed in forest vegetation types at elevations ranging from 700 to 2400 m, while grey snub-nosed monkeys prefer forest vegetation at altitudes ranging from 1600 to 1900 m [60]. Tibetan macaques have a broader habitat range and thus more access to a wider range of food sources, enabling them to eat a greater variety of vegetation and food types than grey snub-nosed monkeys.

In primates, the availability of food resources within the home range, as well as their body size, limits their diet. The body size of primates not only restricts the types of food they can consume, but also influences their preference for concentrated or dispersed food resources [61,62]. Larger primates tend to have a lower quality and variety of food [63]. Tibetan macaques and grey snub-nosed monkeys are two species of primates with comparable body lengths of 51.0 to 71.0 cm, and 63.7 to 69.0 cm [31,64], respectively. However, Tibetan macaques weigh between 12.5 to 20 kg, while grey snub-nosed monkeys weigh only 13.25 to 15.75 kg [31,65]. Together, they consumed 158 genera of plants belonging to 109 families, with only a few being exclusively consumed by one species. Tibetan macaques fed on 8 families and 26 genera, while grey snub-nosed monkeys fed on 8 genera only. Although both species had similar food preferences, their specific diet compositions differed. Nevertheless, since there is no significant difference in the body shape of Tibetan macaques and grey snub-nosed monkeys, the diet compositions of the two species are also not significantly different, except for the exclusive foods particular to each species.

Previous research has shown that primates possess selectivity toward specific types of food based on their nutritional composition [66]. The quality, quantity, composition, and water content of food also affect the food choices of primates [67]. When high-nutrition value food is in abundance, predators choose it over other low-nutrition food items. Even when multiple food items are available, high-nutrition-value food remains the first choice of predators, with little consideration for low-nutrition food items [68]. Primates have nutritional needs that must be met for life activities, such as metabolism; they primarily consume plant-based foods rich in protein, fat, carbohydrates, water, vitamins, and trace elements. A study by Li et al. [69] found that the nutritional composition of most plants varies throughout the year and *Rhinopithecus roxellana* preferred plants with a high protein content and a low cellulose content. In winter, plants with abundances greater than 1 may also be higher in nutritional value; yet, these plants contribute little nutrition to the diet of Tibetan macaques and grey snub-nosed monkeys as these primates feed primarily on a small subset of staple foods. As proposed by the theory of nutritional balance, animals balance their nutrition by mixing different foods and nutrients rather than maximizing the availability of a single nutrient [70]. In winter, food resources are scarce, and to obtain sufficient energy, Tibetan macaques and grey snub-nosed monkeys consume a significant amount of foods with a low nutritional value, leading to a relatively high proportion of food items with an occurrence rate of less than 1 in their respective diets. Since the nutritional components of the primates’ diets were not analyzed, inferences about their foraging strategies were based solely on the type and ratio of plant species they consumed. Therefore, studying the nutritional composition of their diet will be the primary focus of our future research.

### 4.3. Coexistence Mechanism of the Two Primates

The niche width index, which reflects the proportion of species to spatial resources, spatial distribution range, and uniformity [71], was used in this study. The results of this study indicated that Tibetan macaques have a greater advantage in resource competition than grey snub-nosed monkeys since the niche width index of Tibetan macaques was higher. Wang et al. [72] discovered that winter Tibetan macaques and grey snub-nosed monkeys had different temporal and spatial niches, which could minimize the competition for space. Nevertheless, in our study, we found that Tibetan macaques and grey snub-nosed monkeys had almost identical diets, with a food overlap index close to 1. This finding contradicts our original hypothesis (2), which suggests that the coexistence mechanism between the two primates occurs via the differentiation of their temporal and spatial niches rather than via specialization in trophic niches. The results of our study regarding trophic niche overlap contrasted with those from studies focusing on coexisting lowland gorillas and chimpanzees in Gabon and studies on *Saguinus mystax* and *Saguinus fuscicollis* in the Amazon. The different species demonstrate distinct foraging strategies, leading to niche separation [73,74]. Scholars have utilized DNA metagenomics to investigate the dietary overlap between sika deer and wild buffalo within the Northeast China Tiger and Leopard National Park, and the findings indicated a significant degree of dietary convergence in both species [75]. Notably, food resources vary across different study areas. Additionally, each species has a unique foraging preference, which means that different research results are possible.

To a significant extent, the coexistence of species relies on the resource differentiation among them. According to the competitive exclusion theory, coexisting species in the same habitat require exclusive temporal, spatial, and food niches in order to obtain sufficient resources for survival and reproduction [4,76]. While the winter trophic niches of Tibetan macaques and grey snub-nosed monkeys distributed in the FJMR were almost entirely overlapping, the niche breadth of both species throughout other seasons remains unknown. Therefore, it is necessary to conduct additional studies to determine whether feeding niches diverge in spring, summer, and autumn.

## 5. Conclusions

In this study, we utilized DNA metabarcoding to explore the diets of Tibetan macaques and grey snub-nosed monkeys. The results indicated differences in their food consumption. Tibetan macaques had a higher food niche width index than grey snub-nosed monkeys; however, both had a high trophic niche overlap. Therefore, trophic niche differentiation is not the mechanism by which Tibetan macaques and grey snub-nosed monkeys coexist.

## Figures and Tables

**Table 1:** Fecal sampling information.

Group Information	Sample Number
Tibetan macaques	DH1, DH2, DH3, DH4, LJB1, LJB2, EB2, KZB2, KZB4, DH_a, HTP_a, KZB_a, KZB_c, EB_b, LJB_a, LJB_b, LJB_c, LJB_d
Grey snub-nosed monkeys	BP1, BP2, EB1, KZB1, KZB3, BP_c, BP_e, KZB_b, KZB_d, KZB_e, EB_a

**Table 2:** Comparison of food diversity index and niche width between Tibetan macaques and grey snub-nosed monkeys.

	*B*		*D_ij_*	
	Family	Genus	Family	Genus
Tibetan macaques	9.3865	10.2150	0.9998	0.9930
Grey snub-nosed monkeys	8.1951	9.2386		

## Data Availability

The data presented in this study are available upon request from the corresponding author.
